# A method for determination of hematocrit using the mobile app “HaemoCalc”: Validity, reliability, and effect of user expertise

**DOI:** 10.14814/phy2.70314

**Published:** 2025-04-15

**Authors:** Lawrence D. Hayes, Nilihan E. M. Sanal‐Hayes, Maryam Ellam, Marie Mclaughlin, Michelle G. Swainson, Nicholas F. Sculthorpe

**Affiliations:** ^1^ Lancaster Medical School Lancaster University Lancaster UK; ^2^ School of Health and Society University of Salford Salford UK; ^3^ Physical Activity for Health Research Centre, Institute for Sport, P.E. and Health Sciences University of Edinburgh, Moray House School of Education and Sport Edinburgh UK; ^4^ Sport and Physical Activity Research Institute, School of Health and Life Sciences University of the West of Scotland Glasgow UK

**Keywords:** hematocrit, limits of detection, medical education, mobile app, red blood cells, software validation

## Abstract

We evaluated validity, reliability, and effect of user expertise of “HaemoCalc”, a mobile phone application for hematocrit (Hct) measurement from fingerpick blood samples, compared to a traditional Hawksley microhaematocrit reader (MHR). Experiment 1 examined the effect pitch angle during image capture exerted on the validity of Hct values. Twenty participants' samples were analyzed at 0°, 10°, and 20° directly over the sample, and 33° with a 10 cm setback. Analysis of variance (ANOVA) revealed a significant effect of angle on Hct values (*p* < 0.01). Measurements at 33° pitch differed from other angles and the MHR (*p* < 0.001, *d* = 2.31–3.06). Bland–Altman analysis showed good agreement at 0°, 10°, and 20° (mean differences: −0.4% to 1.0%) but poor agreement at 33° (mean difference: −4.4%, LOA: −0.7% to 8.4%). Experiment 2 assessed inter‐ and intra‐rater reliability of expert and novice users (*n* = 12). Participants performed three trials each. HaemoCalc and MHR showed excellent reliability (ICC = 0.95–1.00). No differences were observed between experts and novices (*p* = 1.000, *d* = 0.01–0.39). HaemoCalc is a valid and reliable tool for Hct measurement at small pitch angles and in expert and novice users. The HaemoCalc app offers scalability, repeatability, health and safety benefits, and potential applications in medical education and remote learning.

## 1 INTRODUCTION

Hematocrit (Hct) is the ratio of red blood cells (RBCs) to the total volume of blood and is an important and long‐established physiological marker of circulatory capacity to deliver oxygen to respiring tissues (Blix & Hedin, 1891). Low hematocrit, or anemia, can be caused by reduced production of RBCs or an increase in destruction of RBCs, with symptoms manifesting as dyspnoea, dizziness, headache, cold, pale skin, and chest pain (Balarajan et al., 2011). This can occur in conditions of malnutrition (Gonzalez‐Casas et al., 2009), cardiovascular disease (Kishimoto et al., 2020), diabetes mellitus (Irace et al., 2011; Koponen et al., 2013; Wu et al., 2018), blood cancer and disorders (Gilleece et al., 2020), HIV (Omoregie et al., 2008), and liver cirrhosis (Gonzalez‐Casas et al., 2009). This is valuable as the accurate measurement of Hct is a routine part of any hematology assessment. Though once a mainstay of routine hematology assessment, Hct measurements have become less common in clinical practice, as broader blood panels and other biomarkers now offer more comprehensive patient assessments. As a result, Hct is sometimes seen as a “forgotten measure” in healthcare, despite its value in contexts requiring quick, straightforward assessment of oxygen‐carrying capacity—such as exercise physiology, altitude adaptation studies, or chronic disease monitoring (Mondal & Zubair, 2024).

Traditional Hct readers can measure Hct to an accuracy of approximately 0.5%; however, this limited accuracy can be affected by minor misalignments such as the Hct reader line being angled through the center of the interface, potentially introducing subtle errors. A digital system overcomes this as it relies on pixel density for measurements, allowing for accuracy levels within 0.1% in most cases. The digital line reader is horizontally aligned through the buffy coat, minimizing issues with sample misalignment, and users can magnify the image to enhance precision. Another drawback of traditional Hct readers is that the sample is discarded after measurement, meaning there is no possibility for retrospective assessment if issues like over‐ or under‐spinning are suspected. This also prevents double‐checking or future re‐evaluation of measurements, limiting inter‐rater reliability (Furth, 1956). In contrast, a digital image could serve as a record allowing for re‐analysis or further inspection if necessary, such as for evidence of haemolysis or other artifacts. Traditional Hct readers lack automated or digital data capture, making it challenging to integrate directly with data systems—a feature that could be incorporated into app‐based solutions for streamlined data management. Another key advantage of digital measurement is that it reduces the risk of sample contamination as it does not require handling during measurement, potentially reducing biohazard exposure for the user.

Digital measurement of Hct has some advantages, as described above. Additionally, there has been a spread of the “lab in the pocket” concept in recent years (Mather et al., 2024; Mehta et al., 2017; Perkel, 2017; Radin et al., 2016; Stampfer et al., 2020). Smartphone usage is on the rise worldwide, can permeate remote regions, and operates with relatively low infrastructure requirements. Smartphones have powerful computational potential and require minimal user training. Thus, mobile health (mHealth) has several advantages over traditional methods of measurement (Free et al., 2013; Mair et al., 2022, 2023). mHealth can improve health care access, permit self‐monitoring of vital signs, provide automated notifications of specific biometric values, and increase scale and reach of research through reduced participant burden associated with travel (Amelard et al., 2015; Meach et al., 2024; Perez et al., 2019; Pluymaekers et al., 2021; Sculthorpe et al., 2025). In 2020 we developed a bespoke mobile app to measure Hct, and subsequently named this app the “HaemoCalc” app (Sculthorpe, 2020). Although we originally developed the HaemoCalc app for remote teaching purposes during the COVID‐19 pandemic, we considered it pragmatic to validate the HaemoCalc app for future use where validity and reliability is important (i.e., research purposes). As such, the aim of this study was to validate the HaemoCalc app against the Hawksley MHR for the determination of Hct. A secondary aim was to evaluate the inter‐ and intra‐rater reliability and effect of user expertise.

## 2 METHODS

To test the validity of HaemoCalc, we tested the effect of image capture at 0° pitch, 10° pitch, and 20° pitch, and 10 cm setback from the capillary tube with 33° (experiment 1). To test the reliability of HaemoCalc, we determined the inter‐rater and intra‐rater reliability of the HaemoCalc app against the MHR (experiment 2). Finally, we compared the values of novice users (penultimate year sport and exercise student undergraduate students) with an expert user (LDH; first author), with 12 years of post‐doctoral experience (experiment 2).

All participants were provided with a detailed information sheet and signed an informed consent form prior to their involvement. All data collected has been anonymized to ensure participant confidentiality and privacy. This study was approved by the University of West of Scotland Health and Life Sciences Institutional Ethics Review Board.

### 2.1 Experiment 1: Validity

#### 2.1.1 Participants

Twenty participants were recruited for this study, comprising 12 females and 8 males. The sample included a mix of university students and staff. Capillary blood samples were obtained via finger‐prick using a disposable lancet. The collected samples were drawn into heparinized microhaematocrit capillary tubes (Hawksley, Sussex, UK). These tubes were subsequently centrifuged (Hawksley, Sussex, UK) for 3 min at 12,000 revolutions per minute. All samples were analyzed by the same researcher (ME) for consistency. One sample was collected per participant for analysis.

#### 2.1.2 Microhaematocrit reader (MHR)

Hematocrit was measured using a microhaematocrit reader (Hawksley, Sussex, UK) following standard procedures (Hayes & Morse, 2010; van Beaumont et al., 1972).

#### 2.1.3 HaemoCalc hematocrit analyser

**FIGURE 1:**
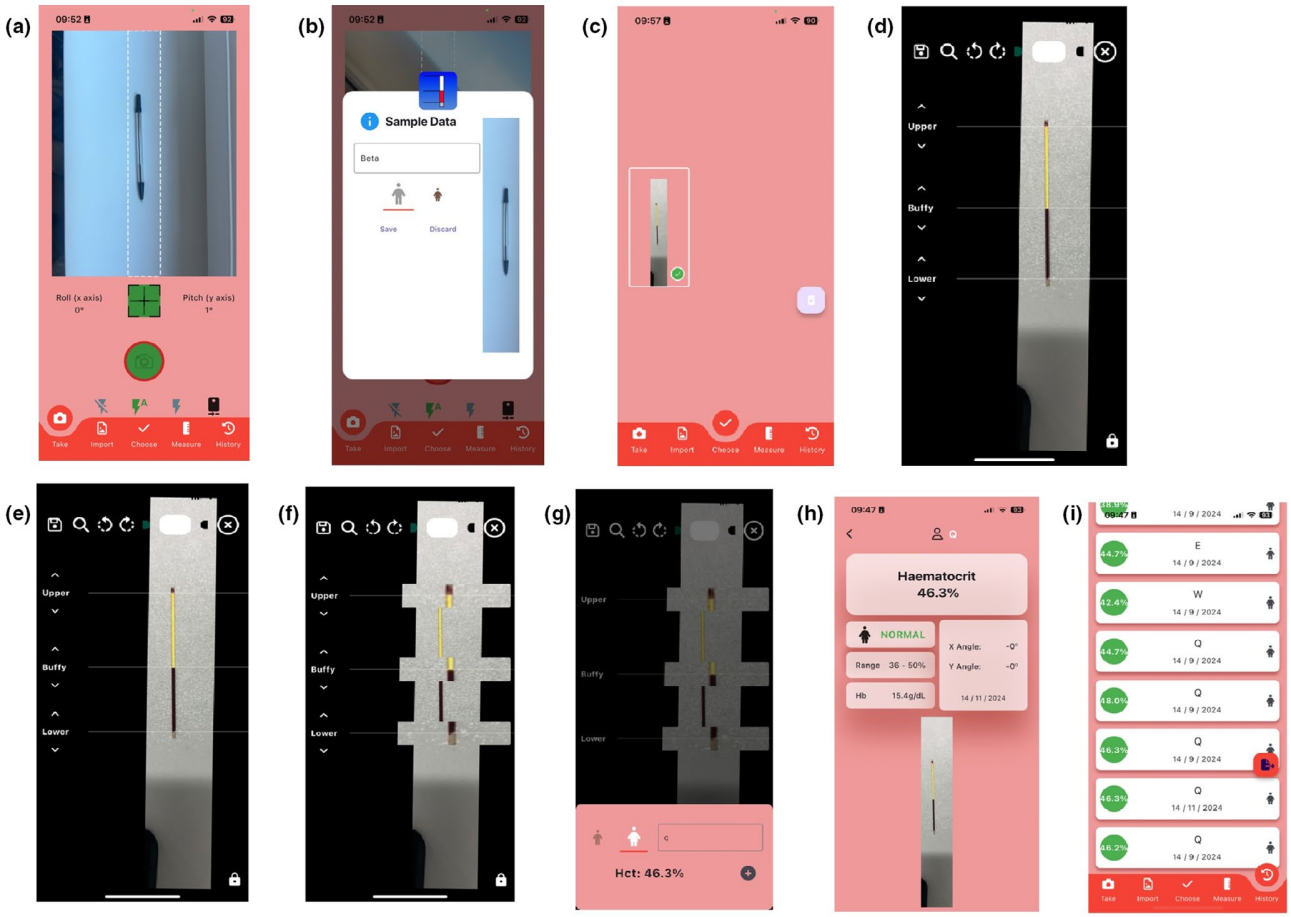
Hematocrit App Usage. (a) An initial image is captured in the HaemoCalc app. (b) Image cropped and sample ID and sex requested. (c) Image imported into the HaemoCalc app from the phone's camera roll (d) The image is adjusted to straighten the capillary tube on the screen and maximize the height on the screen. (e) The draggable bars “Lower”, “Buffy”, and “Upper” are placed in line with the top of the plug, the plasma interface, and the top of the sample, respectively. (f) Sliders turn opaque to aid accuracy. (g) One measured sample information is added, and the Hct value is calculated. (h) The sample data are saved. (i) Previous samples and the associated image can be reviewed, re‐analyzed and exported to Microsoft Excel.

A prototype version of the HaemoCalc application developed for both iOS and Android‐based mobile phones was used for measurement of Hct. A standard camera tripod was used to capture images of the samples at specified pitch angles for reliability. The tripod was positioned at a fixed angle to ensure the iPhone 14 remained stationary throughout the process. The device was 19.5 cm above the capillary tube, which was the upper limit of the tripod. Samples were placed within the camera's field of view to ensure accurate measurement. Images were captured from directly above the capillary tube at 0° pitch, 10° pitch, and 20° pitch, and 10 cm setback from the capillary tube with 33° pitch. These orientations were selected to mimic common locations users may photograph the capillary tubes from, thereby allowing us to assess the HaemoCalc app's performance under real‐world conditions. The final condition (10 cm setback from the capillary tube with 33° pitch) was chosen to mimic the scenario where individuals could be stood back from the desk the capillary tube is placed upon. 33° pitch was required to centre the capillary tube during image capture when 10 cm back from the sample. Once opened, HaemoCalc requested access to the phone's camera, with instructions to take a picture of the sample with the phone as close to parallel to the sample as possible. The capture screen contained a central rectangular section in which the image of the capillary tube was aligned (Figure 1a). The screen also incorporated a reticule to guide the user to have the phone directly over the sample with a visual indicator. The indicator would turn green if the phone had <5° pitch (angled from top‐to‐bottom) and <5° yaw (angled from side‐to‐side). This was incorporated to prompt users that the image capture position was correct. Given our experimental aim, these prompts were ignored for experiment 1 but were used in experiment 2. Once captured images were automatically cropped to the central rectangular region and a sample ID and participant sex was provided, (Figure 1b), after which the image and meta data was stored on the device. Once taken, the anonymised photograph was stored in the phone's internal storage (Figure 1c), and the HaemoCalc app imported it into the main assessment screen (Figure 1d). The assessment screen included several features. In case of skewed images, left and right rotation buttons allow 0.5°adjustments of the image to ensure that the capillary tube was positioned vertically on the screen (Figure 1e). To improve accuracy, “pinch‐to‐zoom” could be performed by the user to ensure the distance from the top of the plug to the top of the sample within the tube was as large as possible on the screen. During this function, rotation was disabled to maintain the vertical position of the sample on the screen. The screen also included three “draggable” touch targets labeled “lower”, “buffy”, and “upper”, each with thin lines extending across the rest of the screen (Figure 1f). Hematocrit was measured by dragging the “lower” slider so that the extended line was aligned with the interface between the top of the clay plug and the red blood cell column. The “buffy” slider was positioned at the interface of the blood and plasma, at the level of the maximum excursion of any concave (or convex) meniscus. Finally, the “upper” slider was positioned at the top of the plasma. During drag operations, the slider label and extended lines became opaque to aid accurate placement. Selector buttons changed the color of the draggable label and line to ensure sufficient contrast with the image. Finally, to aid accurate placement a zoom slider was available to increase magnification around the draggable sliders (Figure 1f). Once the user was satisfied with the measurement, a “save” icon was pressed. At this point, the internal logic of the HaemoCalc app calculates the number of vertical pixels on the screen, the number of pixels between the “lower” and “upper” slider, and the number of pixels between the “buffy” and both “lower” and “upper” slider, and subsequently calculated the proportion of the whole blood (pixels between “lower” and “buffy” slider) relative to the whole sample (pixels between “lower” and “upper” sliders; Figure 1g). This calculation was then recorded as the sample Hct, measured in percentage (Figure 1h). The sample ID, sex, and Hct were stored as a single sample record in an internal database for later recall and export to Microsoft Excel (Figure 1i).

### 2.2 Experiment 2: Reliability and effect of user expertise

#### 2.2.1 Participants

Twelve undergraduate students, of whom three were female, participated as part of a credit‐bearing module on exercise physiology. Students were in their penultimate year of a Sport and Exercise Science bachelor's programme. Hct measurement was undertaken as outlined in study 1. Three trials were performed by each rater for the MHR, and three trials were performed by each rater for the HaemoCalc (i.e., six measures in total, except the expert user who measured each sample six times [72 measurements]).

### 2.3 Statistical analysis

Data were tested for normal distribution by Shapiro–Wilk's test and for homogeneity of variance using Levene's test. For experiment 1, repeated measures analysis of variance (ANOVA) was conducted, followed by post hoc paired sample *t*‐tests with Bonferroni corrections. Bland and Altman (1986) plots were generated to display the mean difference between pitch angles and limits of agreement (LOA) calculated as mean ± 1.96 of the difference between raters or methods. To compare methods, Pearson's product–moment correlation coefficients were calculated to assess relationship between MHR and HaemoCalc values. For experiment 2, a two‐way (user [novice, expert] × method [MHR, HaemoCalc]) ANOVA was conducted, followed by post hoc paired sample *t*‐tests with Bonferroni corrections. Reliability was described using intraclass correlation coefficient (ICC) and the typical error. Bland and Altman (1986) plots were generated to display the mean difference between methods and LOA. Intra‐rater reliability was assessed comparing the three trials by the same measurer. For interrater reliability, definitive Hct values from the novice and the expert measurer for the same blood sample were compared. Moreover, Hct values for both novice and expert measurers compared the MHR and the HaemoCalc. To compare methods, Pearson's product–moment correlation coefficients were calculated to assess the relationship between MHR and HaemoCalc values in novices and in experts. In all instances, we report alpha levels as exact *p* values, without dichotomous interpretation of “significant” or “nonsignificant” as advised by the American Statistical Association (Hurlbert et al., 2019). Effect sizes are reported using partial eta squared (*η*p^2^) for ANOVA and Cohen's *d* (difference in means ÷ pooled standard deviation [SD]) for pairwise comparison. *η*p^2^ was interpreted as small (0.02), medium (0.13), and large (0.26) effects. Cohen's *d* was interpreted as small (0.2), medium (0.5), and large (0.8) effects (Lakens, 2013). Bland‐Altmans were interpreted as good if mean differences were <1.2% because 1.2% of a typical Hct (assuming a typical value of 45%) is 0.5% in Hct. We argue the MHR is unable to detect differences of less than 0.5%, so this is within the threshold of observable differences. All data analyses were conducted in Jamovi (version 2.0), and figures were generated in GraphPad Prism (GraphPad Prism 8.4.3, GraphPad Software Inc., USA) which visualize data as grouped dot plots as recommended (Drummond & Vowler, 2012; Weissgerber et al., 2015). Data are presented in text as mean ± SD.

## 3 RESULTS

### 3.1 Experiment 1: Validity

**FIGURE 2:**
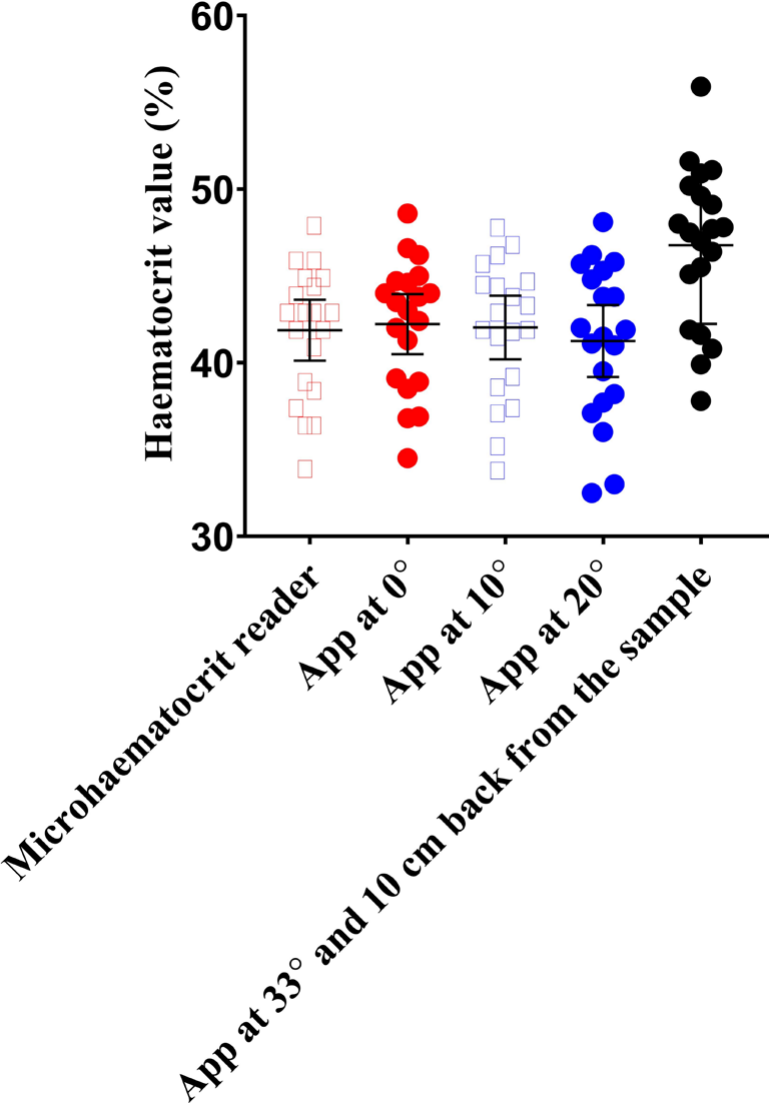
Hematocrit values, in a group of students and staff members, measured using a microhaematocrit reader and a mobile app directly above the sample with a 0° pitch, 10° pitch, 20° pitch, and 10 cm set back from the sample with a 33° pitch. Data are presented as mean ± 95% CI, plus individual data points.

Figure 2 shows data from the HaemoCalc app at several angles and the MHR as a reference value. Individual data points are shown to visualize the spread of data. Repeated measures ANOVA resulted in an effect of pitch angle of *p* < 0.01 (*η*p^2^ = 0.793). The Hct values were 41.9 ± 0.8%, 42.2 ± 0.8%, 42.0 ± 0.9%, 41.2 ± 1.0%, and 46.8 ± 1.0% for the MHR and HaemoCalc app with 0° pitch, 10° pitch, 20° pitch, and 33° pitch with a 10 cm set back, respectively. Post hoc pairwise comparisons resulted in a difference between 33° with a 10 cm set back and the MHR (*p* < 0.001, *d* = 2.33) and app with 0° pitch (*p* < 0.001, *d* = 2.31), 10° pitch (*p* < 0.001, *d* = 2.62), and 20° pitch (*p* < 0.001, *d* = 3.06). A positive Pearson's product–moment correlation existed between MHR values and HaemoCalc app at 0° pitch values (*r* = 0.992, *p* < 0.001).

**FIGURE 3:**
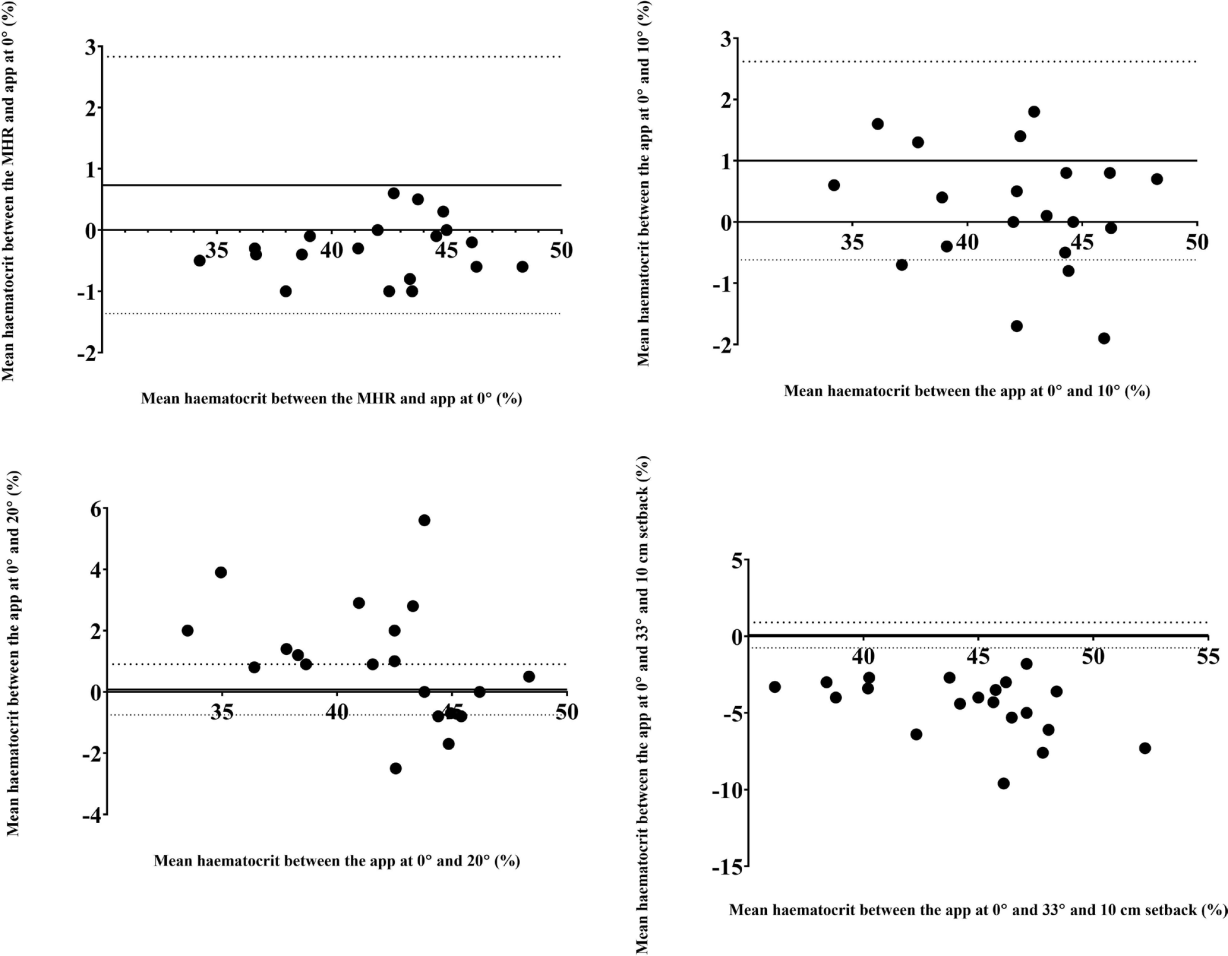
Bland and Altman plot of hematocrit values. Top left: Measured using a microhaematocrit reader (MHR) and HaemoCalc with 0° pitch; Top right: Measured using HaemoCalc with 0° pitch and HaemoCalc with 10° pitch; Bottom left: Measured using HaemoCalc with 0° pitch and HaemoCalc with 20° pitch; Bottom right: Measured using HaemoCalc with 0° pitch and HaemoCalc with 33° pitch and 10 cm setback from the sample. Mean difference is shown as a solid line and limits of agreement (LOA) are dashed lines.

Bland–Altman analysis suggested a good agreement between the MHR and HaemoCalc 0° pitch with a mean difference of −0.35% and LOA of −1.30% to 0.61% (Figure 3). The 10° angle had good agreement with 0° pitch (mean difference of 0.20% and LOA of −1.78% to 2.17%). The 20° angle had good agreement with 0° pitch (mean difference of 0.97% and LOA of −2.79% to 4.73%). The 33° angle had poor agreement with 0° pitch (mean difference of −4.55% and LOA of −0.69% to 8.41%).

### 3.2 Experiment 2: Reliability and effect of user expertise

**FIGURE 4:**
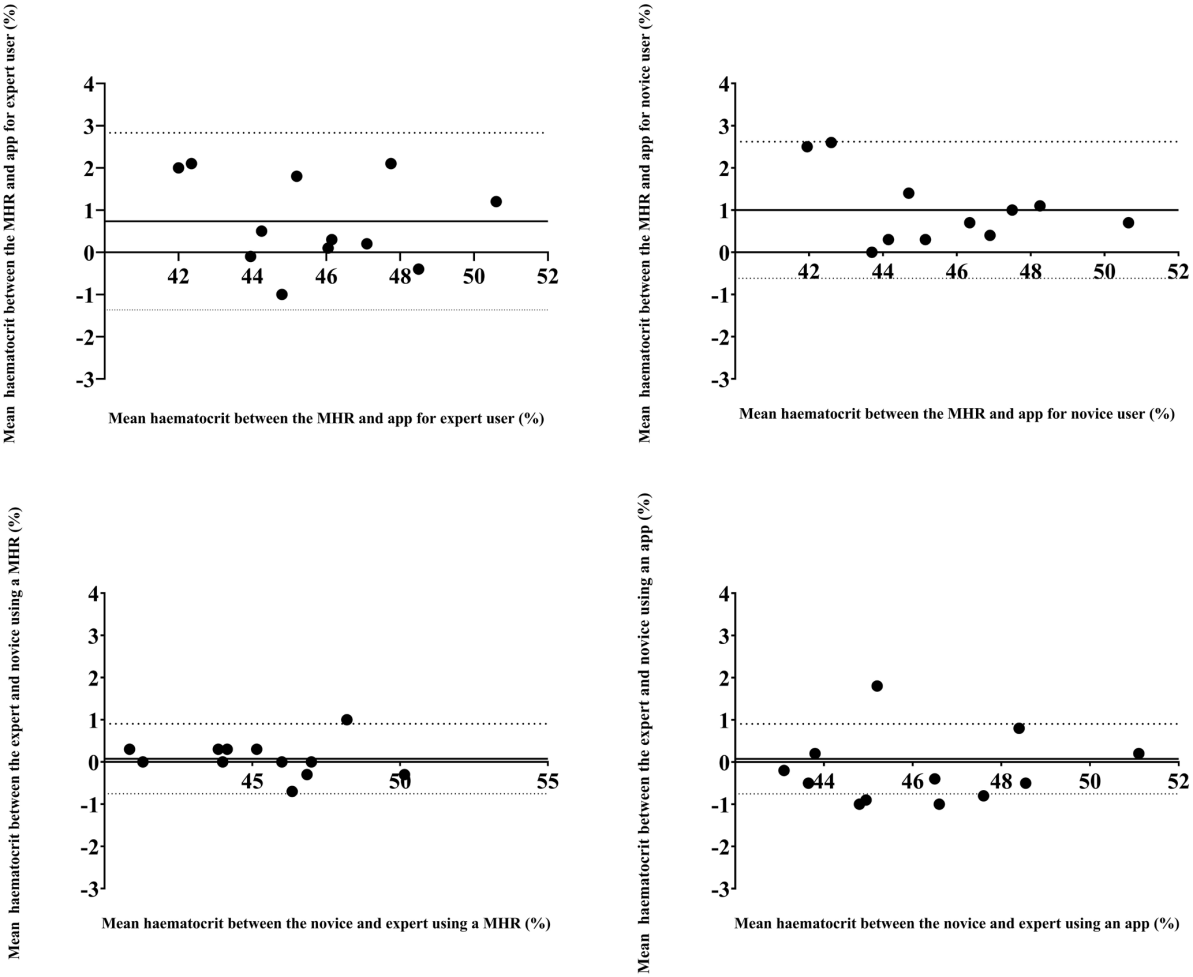
Hematocrit values, in a group of collegiate students, measured using a microhaematocrit reader and a mobile app by an expert and by novices. Data are presented as mean ± 95% CI, plus individual data points.

The ANOVA resulted in no effect of user expertise, method, or interaction (*p* = 0.938, *η*p^2^ = 0.000; *p* = 0.249 *η*p^2^ = 0.030; and *p* = 0.858, *η*p^2^ = 0.001, respectively). The Hct values were 45.4 ± 2.7, 46.1 ± 2.5, 45.3 ± 2.7, and 46.3 ± 2.4% for expert MHR, expert HaemoCalc, novice MHR, and novice HaemoCalc, respectively (Figure 4). Pairwise comparisons resulted in no differences between the four groupings (*p* = 1.00 for all, *d* = 0.01–0.39).

The MHR showed excellent relative intra‐rater reliability (novice ICC = 0.99 [95% CI = 0.96–1.00], typical error = 0.17; expert ICC = 0.98 [95% CI = 0.95–0.99], typical error = 0.06), as did HaemoCalc (novice ICC = 0.0.97 [95% CI = 0.91–0.99], typical error = 0.17; expert ICC = 1.00 [95% CI = 1.00–1.00], typical; error = 0.06). The MHR and HaemoCalc both showed excellent relative inter‐rater reliability (MHR ICC = 0.99 [95% CI = 0.97–1.00], typical error = 0.11; app ICC = 0.95 [95% CI = 0.87–0.98], typical error = 0.24). A positive Pearson's product–moment correlation existed between expert MHR values and expert HaemoCalc values (*r* = 0.916, *p* < 0.001). A positive Pearson's product–moment correlation existed between novice MHR values and novice HaemoCalc values (*r* = 0.957, *p* < 0.001).

**FIGURE 5:**
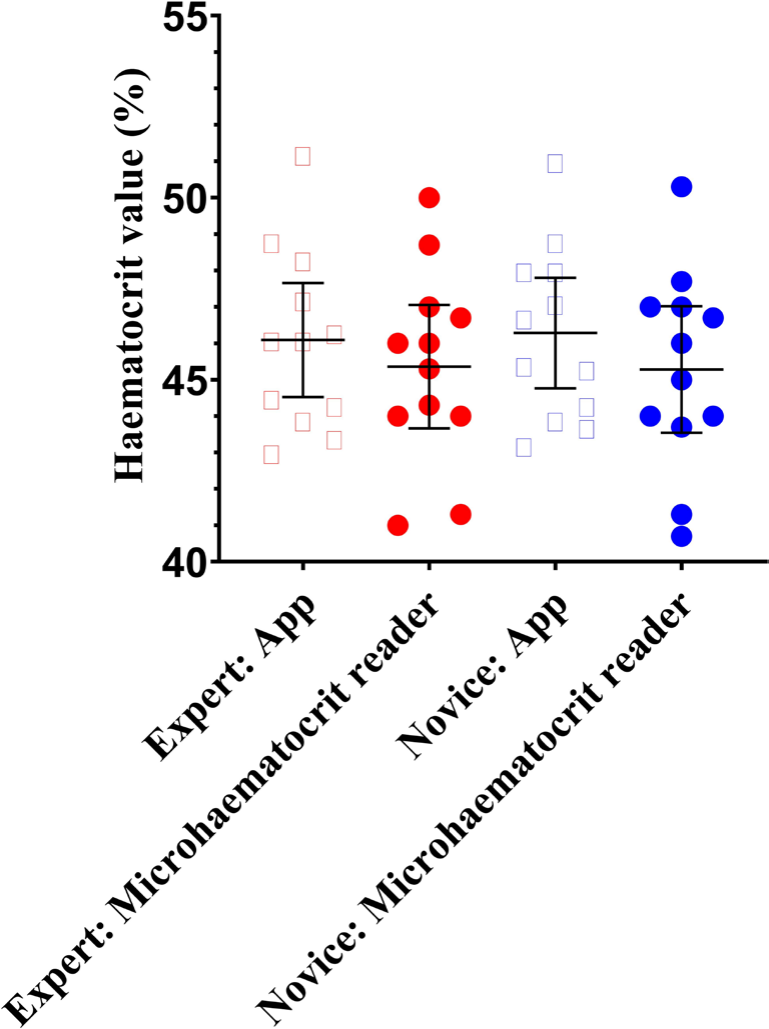
Bland and Altman plot of hematocrit values. Top left: Measured using a microhaematocrit reader (MHR) and HaemoCalc by an expert; Top right: Measured using a microhaematocrit reader (MHR) and HaemoCalc by a novice; Bottom left: Measured using a microhaematocrit reader (MHR) by an expert and novices; Bottom right: Measured using HaemoCalc by an expert and novices. Mean difference is shown as a solid line, and limits of agreement (LOA) are dashed lines.

Bland–Altman analysis suggested a good agreement between the MHR and HaemoCalc with a mean difference of 0.73% and LOA of −1.37% to 2.83% in the expert user (Figure 5). In the novice users, a good agreement between the MHR and HaemoCalc was observed (mean difference of 1.00% and LOA of −0.62% to 2.62%). Bland–Altman analysis resulted in good agreement between expert and novice users for the MHR with a mean difference of 0.08% and LOA of −0.75% to 0.90%. Bland–Altman analysis suggested good agreement between the expert and novice users for HaemoCalc with a mean difference of 0.19% and LOA of −1.83% to 1.45%.

## 4 DISCUSSION

This is the first study of its kind to use a mobile app to measure Hct in centrifuged capillary tubes. We rigorously tested the HaemoCalc app in terms of validity and reliability, and HaemoCalc provides an advancement in streamlining measurement, analysis, and storage of Hct values using technology. The HaemoCalc app builds on previous work using desktop computing (Livshits et al., 2022), and advances the “lab in the pocket” concept (Mehta et al., 2017; Perkel, 2017; Radin et al., 2016; Stampfer et al., 2020) to Hct. Results of this project indicate that HaemoCalc is an accurate and reliable method of assessing Hct in centrifuged capillary tubes and is a suitable method of assessment for both novice and expert assessors. Results from experiment 1 showed good agreement between HaemoCalc at most pitch angles (0°, 10°, and 20°) and the traditional MHR, suggesting the HaemoCalc app can tolerate some deviations from the ideal positioning, which may be beneficial in real‐world settings where precise alignment of the camera may not always be possible. The significant differences seen at the 33° pitch with a 10 cm setback suggest that the HaemoCalc app's accuracy deteriorates with more extreme angles, or when the phone is not directly over the sample. While minor off‐pitch angles are acceptable, extreme angles should be avoided to ensure accurate measurements which is key for guiding future app users in clinical or field settings. Thus, if the instruction to the end user emphasize that images must be captured directly above the capillary tube, some variation in pitch angle seems tolerable in terms of validity. Additionally, as the HaemoCalc app contains a reticule indicator, users are guided to achieve an angle close to 0° as possible, removing guesswork and enhancing accuracy.

Results from experiment 2 demonstrate no differences in hematocrit values between the four data sets: novice HaemoCalc users, expert HaemoCalc users, novice MHR users, and expert MHR users. Moreover, results reveal good inter‐rater reliability between MHR and the HaemoCalc app, between novice and expert users for MHR, and between novice and expert users for the HaemoCalc app. Finally, Bland–Altman analysis demonstrated strong agreement between raters and between methods. Therefore, these findings suggest HaemoCalc is a reliable and valid tool for assessing Hct. This is valuable as the accurate measurement of Hct is a routine part of any hematology assessment.

The ICCs indicate a strong level of agreement with a low mean bias between the two techniques. Similarly, there was also a very low mean bias when comparing novice and expert users (Figure 3), indicating that HaemoCalc maintains the good agreement between novice and expert users seen with MHR measurements. This has important implications for clinical practice and education. In practice, HaemoCalc can be reliably used by healthcare workers with varying expertise levels without compromising accuracy. In education, it acts as a hands‐on tool for students to learn hematocrit measurement and builds confidence in laboratory skills. While the difference between measures is small, it is worth noting some technical differences between the two approaches. MHR scales have the smallest division of 1%, and a visual inspection has a feasible accuracy of 0.5% intervals (e.g., where the measurement line falls between 2 division markers). In contrast, a phone screen enables a more precise measurement. For example, on a relatively small screen such as an iPhone SE, the screen height is 1136 physical pixels, but for compatibility reasons, it groups them into a virtual viewport with a default height of 586 logical pixels. If we assume that when appropriately “pinched to zoom” on screen, the distance from the plug to the top of the plasma occupies 75% of the screen height, then the smallest measurement increment is 0.23% while on larger screens (e.g., iPhone 13 Pro Max with a height of 932 logical pixels), this smallest graduation is 0.1% increments.

Similarly, on an MHR, the measurement line is approximately the width of a single measurement division. Not only does this make judging 0.5% increments difficult, but in novice users, it makes aligning the measurement line with the RBC‐plasma interface more challenging. In contrast, the measurement lines on the HaemoCalc app are much thinner (approx. 5 pixels) and turn opaque when being dragged to aid accurate positioning. Consequently, the ability to measure Hct at twice to five times the resolution allied to thinner and easier‐to‐align markers means that it is inevitable that there will be small differences in measures from the two techniques.

Despite the importance of Hct as a cornerstone of blood analysis, the validity and reliability of different approaches have received less scrutiny than other hematological parameters such as hemoglobin or RBC counts (Bull & Hay, 2001; Gebretsadkan, 2015) limiting the ability to contextualize the results of the present investigation, as does the fact that this appears to be the first attempt to use a mobile phone app to assess Hct. Bull and Hay (2001) assessed the effect of different diameters of capillary tubes on the reliability of manual Hct readers. Of the 12 tubes, 10 had adequate validity when compared with automated methods, with correlation coefficients ranging from 0.976 to 0.962. In the present study, the calculated ICCs are in a similar range (1.00–0.97), suggesting that the level of agreement between the two devices is similar to the acceptable agreement between Hct readers and automated methods. Similarly, Gebretsadkan (2015) compared MHR and automated measurements in a sample of patients. They reported a correlation coefficient of 0.95, again similar to those reported here between MHR and HaemoCalc. It should also be noted that there are known differences between MHR and automated measures, which explains the lower automated values reported by Gebretsadkan (2015). MHR routinely includes a small amount of plasma trapped in the RBC column (England et al., 1972) while some automated methods will ignore RBC agglutinates since they are above the cell volume threshold (Buttarello, 2016). These issues together mean that MHR measures are often larger than automated measures of Hct.

The only previous study using a similar technique to the present work was undertaken by Livshits et al. (2022). These authors compared the use of automated Hct assessment, MHR, and their own using an image processing technique. Like our approach, they used computer image software (ImageJ, NIH, USA) to measure the number of screen pixels between the capillary tube plug and buffy coat as a proportion of the pixels between the plug and the top of the plasma column. Their correlation coefficient between MHR and their technique was very similar to the ICC findings reported here (0.965). They concluded that their approach is a valid and reliable method that removes many of the sources of error inherent to standard MHR use. Our study extends this by standardizing image capture using on‐screen feedback from phone sensors and simplifying the steps between the acquisition of a Hct image and its measurement since they all occur within HaemoCalc.

There are some limitations of the present study that should be noted. First, our aim was to replicate “real‐world” use of HaemoCalc. Correspondingly, while we took care to take pictures from a plane directly above the sample, and encouraged novice participants to do likewise, we did not use any additional features (such as crosshairs) built into the phone to ensure that we minimized any parallax error. However, it seems likely that a more standardized approach to camera position during image acquisition would improve rather than reduce reliability. Nevertheless, future work should aim to quantify the degree of error that different camera angles might introduce. Future versions of HaemoCalc could also use the phone sensors to identify to the user when the camera is horizontal (i.e., when phone pitch and yaw are both zero) above the blood sample. In this context, we do not know the effect of increased yaw or roll angle, and this requires further study. Second, we limited our analysis to healthy participants only, and therefore our measurement samples were all within the “normal” range for adult males and females. Again, future work could examine if HaemoCalc measures of Hct at either extreme (e.g., anemia or polycythaemia) are equally valid.

Additionally, participants in experiment 2 were undergraduate students and technologically literate (in terms of mobile devices). It is possible that other populations may have had more difficulty using the HaemoCalc. However, our anecdotal observations were that our participants had more difficulty using the MHR than HaemoCalc, and even more difficulty obtaining a capillary blood sample than using either HaemoCalc or the MHR. Camera quality may vary between devices (and thus users), although this should be mitigated since measures are taken from the image as rendered on screen rather than from the underlying image file directly. Finally, an important issue to note is the financial commitment associated with the purchase of a mobile device, which may, in turn, be affected by socioeconomic status. We have attempted to mitigate this issue as far as possible, making the HaemoCalc app available on as wide a range of devices as possible (including iOS and Android devices). Nevertheless, the idea that using a mobile device will help assessments in low‐resource settings may not always be the case. In this context, other accessibility factors, such as sensory impairment, dexterity issues, or any specific conditions that influence app usability, must be considered, though they are likely to affect MHR measurements equally. A limitation of the HaemoCalc app is that accurate hematocrit measurement requires the skill to take blood and access to a centrifuge; therefore, future work could explore the possibility of manual centrifugation methods to make hematocrit testing more accessible.

While the sample size was appropriate for the study, larger studies would be useful to confirm these findings across different populations and environments. Any limitations related to environmental conditions (e.g., lighting and background) might influence the accuracy of the HaemoCalc app in real‐world field applications. Further validation of HaemoCalc under different clinical conditions (e.g., in varying light conditions) is needed. Testing the HaemoCalc app in more diverse and less controlled environments would help to determine its robustness and utility in more real‐world scenarios.

In addition to these limitations, there are some benefits to this technique that should also be noted. First, and as outlined, this project developed out of the need to develop remote methods for teaching. In this regard, the introduction of HaemoCalc was successful, enabling students to measure multiple samples with different characteristics despite remote teaching during the COVID‐19 lockdowns. Secondly, and in line with the “lab in the pocket” concept (Radin et al., 2016; Stampfer et al., 2020) each student or researcher was able to have a version of HaemoCalc on their device rather than waiting for access to one of a limited number of physical MHR devices to become available. Thirdly, measurement results are stored through a streamlined process in an easy‐to‐access manner and exportable to a standard spreadsheet. A fourth benefit, and with specific reference to Hct measurement of samples collected by novices, HaemoCalc can “rescue” poorer quality samples. For example, the MHR requires sufficient blood volume in the capillary tube to anchor the 0% and 100% values to, whereas when using digital image files, users can zoom to fit the capillary tube to the screen size. While the assessment of small samples in research or clinical settings may result in erroneous results, in a teaching setting it can help students stay engaged with laboratory sessions. Fifthly, it reduces risk of contamination as users can capture an image of the sample in the centrifuge incision. Finally, the samples are numbered to aid later identification, and the image of the sample is not destroyed, and the sample data is linked to the image in the camera roll, allowing for later repeat assessment (e.g., if haemolysis is suspected, or if the data is lost).

## 5 CONCLUSION

HaemoCalc is a valid and reliable method of assessing Hct if images are captured directly above the sample, with excellent inter‐ and intra‐rater reliability. Moreover, good agreement between the two user groups (novice and expert) and between the two measurement tools (MHR and HaemoCalc) was observed. This approach simplifies the processing of samples with a greater measurement resolution than is possible with standard MHR assessment while also reducing the risk of contamination and allows for later reanalysis of the sample.

## AUTHOR CONTRBUTIONS

Nicholas F Sculthorpe was involved in conceptualization and resources. Lawrence D Hayes and Nicholas F Sculthorpe were involved in methodology. Lawrence D Hayes, Nilihan EM Sanal‐Hayes, Maryam Ellam, Marie Mclaughlin, Michelle G Swainson, and Nicholas F Sculthorpe were involved in formal analysis, investigation, writing—original draft preparation, writing—review and editing. Lawrence D Hayes was involved in visualization and project administration. Lawrence D Hayes, Michelle G Swainson, and Nicholas F Sculthorpe were involved in supervision. Lawrence D Hayes and Michelle G Swainson were involved in funding acquisition.

## FUNDING INFORMATION

No sources of funding were utilized for this work.

## CONFLICT OF INTEREST STATEMENT

All authors declare that they have no conflicts of interest.

## ETHICS STATEMENT

Ethical approval was granted by the University of Scotland Institutional Ethics Pannel.

## Data Availability

Data are available here: https://www.dropbox.com/scl/fi/zuf6y06kz3v60cwx2xj85/HaematApp‐data‐sheet‐OPEN.xlsx?rlkey=91h3srbhguiud7pw0kwkhwdl8&st=1y15qtf5&dl=0.
